# Open-chest cardiopulmonary resuscitation versus closed-chest cardiopulmonary resuscitation in patients with cardiac arrest: a systematic review and meta-analysis

**DOI:** 10.1186/s13049-019-0690-7

**Published:** 2019-12-27

**Authors:** Mao Wang, Xiaoguang Lu, Ping Gong, Yilong Zhong, Dianbo Gong, Yi Song

**Affiliations:** 1 Emergency Department, Affiliated Zhongshan Hospital of Dalian University, Dalian city, Liaoning Province China; 2 Emergency Department, First Affiliated Hospital of Dalian Medical University, Dalian, 116011 China

**Keywords:** Closed-chest cardiopulmonary resuscitation, Open-chest cardiopulmonary resuscitation, Blunt trauma, Systematic review, Meta-analysis

## Abstract

**Background:**

Cardiopulmonary resuscitation is the most urgent and critical step in the rescue of patients with cardiac arrest. However, only about 10% of patients with out-of-hospital cardiac arrest survive to discharge. Surprisingly, there is growing evidence that open-chest cardiopulmonary resuscitation is superior to closed-chest cardiopulmonary resuscitation. Meanwhile, The Western Trauma Association and The European Resuscitation Council encouraged thoracotomy in certain circumstances for trauma patients. But whether open-chest cardiopulmonary resuscitation is superior to closed-chest cardiopulmonary resuscitation remains undetermined. Therefore, the aim of this study was to summarize current studies on open-chest cardiopulmonary resuscitation in a systematic review, comparing it to closed-chest cardiopulmonary resuscitation, in a meta-analysis.

**Methods:**

In this systematic review and meta-analysis, we searched the PubMed, EmBase, Web of Science, and Cochrane Library databases from inception to May 2019 investigating the effect of open-chest cardiopulmonary resuscitation and closed-chest cardiopulmonary resuscitation in patients with cardiac arrest, without language restrictions. Statistical analysis was performed using Stata 12.0 software. The primary outcome was return of spontaneous circulation. The secondary outcome was survival to discharge.

**Results:**

Seven observational studies were eligible for inclusion in this meta-analysis involving 8548 patients. No comparative randomized clinical trial was reported in the literature. There was no significant difference in return of spontaneous circulation and survival to discharge between open-chest cardiopulmonary resuscitation and closed-chest cardiopulmonary resuscitation in cardiac arrest patients. The odds ratio (OR) were 0.92 (95%CI 0.36–2.31, *P* > 0.05) and 0.54 (95%CI 0.17–1.78, *P* > 0.05) for return of spontaneous circulation and survival to discharge, respectively. Subgroup analysis of cardiac arrest patients with trauma showed that closed-chest cardiopulmonary resuscitation was associated with higher return of spontaneous circulation compared with open-chest cardiopulmonary resuscitation (OR = 0.59 95%CI 0.37–0.94, *P* < 0.05). And subgroup analysis of cardiac arrest patients with non-trauma showed that open-chest cardiopulmonary resuscitation was associated with higher ROSC compared with closed-chest cardiopulmonary resuscitation (OR = 3.12 95%CI 1.23–7.91, *P* < 0.05).

**Conclusions:**

In conclusion, for patients with cardiac arrest, we should implement closed-chest cardiopulmonary resuscitation as soon as possible. However, for cardiac arrest patients with chest trauma who cannot perform closed-chest cardiopulmonary resuscitation, open-chest cardiopulmonary resuscitation should be implemented as soon as possible.

## Introduction

Cardiopulmonary resuscitation (CPR) is the most urgent and critical step in the rescue of patients with cardiac arrest [1]. It provides a second chance to many patients with cardiac arrest. When Kouwenhoven described closed-chest cardiopulmonary resuscitation (CCCPR) in detail in 1960 with a related 70% long-term survival rate, open-chest cardiopulmonary resuscitation (OCCPR) was marginalized from the mainstream practice, and CCCPR became the preferred method of resuscitation [2–4]. Today, however, the success rate of CCCPR remains far below expectations. The rate of survival to discharge in patients with out-of-hospital cardiac arrest is only 10% worldwide [5]. And the survival rates of out-of-hospital cardiac arrest in Asia, North America, Europe, and Australia are 2, 6, 9, and 11%, respectively [6].

The low survival rate of patients with cardiac arrest can be attributed to many factors. An important variable determining survival in cardiac arrest is whether adequate circulation can be restored within a limited time [7, 8]. Although chest compression increases coronary perfusion pressure and also delivers blood to the vital organs such as brain, studies showed that myocardial perfusion and cardiac output produced by chest compression only constitute a small fraction of normal blood flow [9–12].

Meanwhile, multiple studies have consistently demonstrated that open chest cardiac massage produces higher coronary perfusion pressure and elevated systemic flow compared with closed chest cardiac massage [13]. It may also facilitate return of spontaneous circulation (ROSC) and survival [14–16]. The Western Trauma Association and The European Resuscitation Council encouraged thoracotomy in certain circumstances for trauma cardiac arrest patients [17, 18].

But whether OCCPR is superior to CCCPR remains undetermined. Therefore, the aim of this study was to summarize current studies assessing OCCPR in a systematic review, comparing it to CCCPR in a meta-analysis.

## Methods

### Data sources and search strategy

The MOOSE (Meta-analysis Of Observational Studies in Epidemiology) [19] guidelines were followed in this systematic review and meta-analysis. And the selection of studies was done by the PRISMA (Preferred Reporting Items for Systematic Reviews and Meta-Analysis) 2009 Statement. The PubMed, EMBASE, Web of Science, and Cochrane Library databases were searched from inception to May 2019 for all relevant studies. The following search terms were used: “cardiac arrest”, “open-chest cardiopulmonary resuscitation”, “open chest cardiac massage”, “internal cardiac massage”, “open direct cardiac massage”, “closed-chest cardiopulmonary resuscitation”, “closed chest cardiac massage”, “standard cardiopulmonary resuscitation”, “cardiopulmonary resuscitation”. No language restrictions were imposed.

### Selection criteria

Studies included in this meta-analysis fulfilled the following criteria (PICOS): (1) participants, patients with cardiac arrest due to any causes; (2) intervention, OCCPR; (3) comparisons, CCCPR; (4) outcomes, detailed information for ROSC or survival to discharge available; (5) study design, observational studies comparing OCCPR and CCCPR for their effects in patients with cardiac arrest, no comparative randomized clinical trial was reported in the literature.

Studies were excluded if they were case reports, conference or poster abstracts, reviews, letters, or articles not containing original data.

### Outcomes

The primary outcome of the current meta-analysis was ROSC, defined as spontaneous palpable pulse. The Secondary outcome was survival to discharge.

### Data extraction and quality assessment

Data were extracted from each eligible study by 3 reviewers independently, using data extraction table designed by the authors, including year of publication, first author’s name, study design, study period, patient characteristics, sample size, type of intervention and outcomes. Any disagreements were resolved by consensus. The risk of bias was assessed by The Newcastle-Ottawa Scale (NOS) for observational studies, which included three determinants of quality: selection, comparability, and exposure or outcome assessment. The maximum scores were 4 points for selection, 2 for comparability and 3 for outcome. A total of 6–9 points indicated high quality. The highest score was 9 points, reflecting the highest quality.

### Quantitative analysis

The meta-analyses were performed with Stata 12.0. Odds ratio (OR) and 95% confidence interval (CI) were computed for binary variables. Heterogeneity was quantitatively evaluated by I^2^ statistic (no heterogeneity, I^2^ = 0–25%; moderate heterogeneity, I^2^ = 25–50%; large heterogeneity, I^2^ = 50–75%; extreme heterogeneity, I^2^ = 75–100%). The random effects model was used for I^2^ > 50%; otherwise, the fixed effects model was employed. Potential publication bias was examined by the funnel plot, whose asymmetry was examined by Begg’s tests. *P* < 0.05 indicated publication bias.

### Study selection

**Fig. 1:**
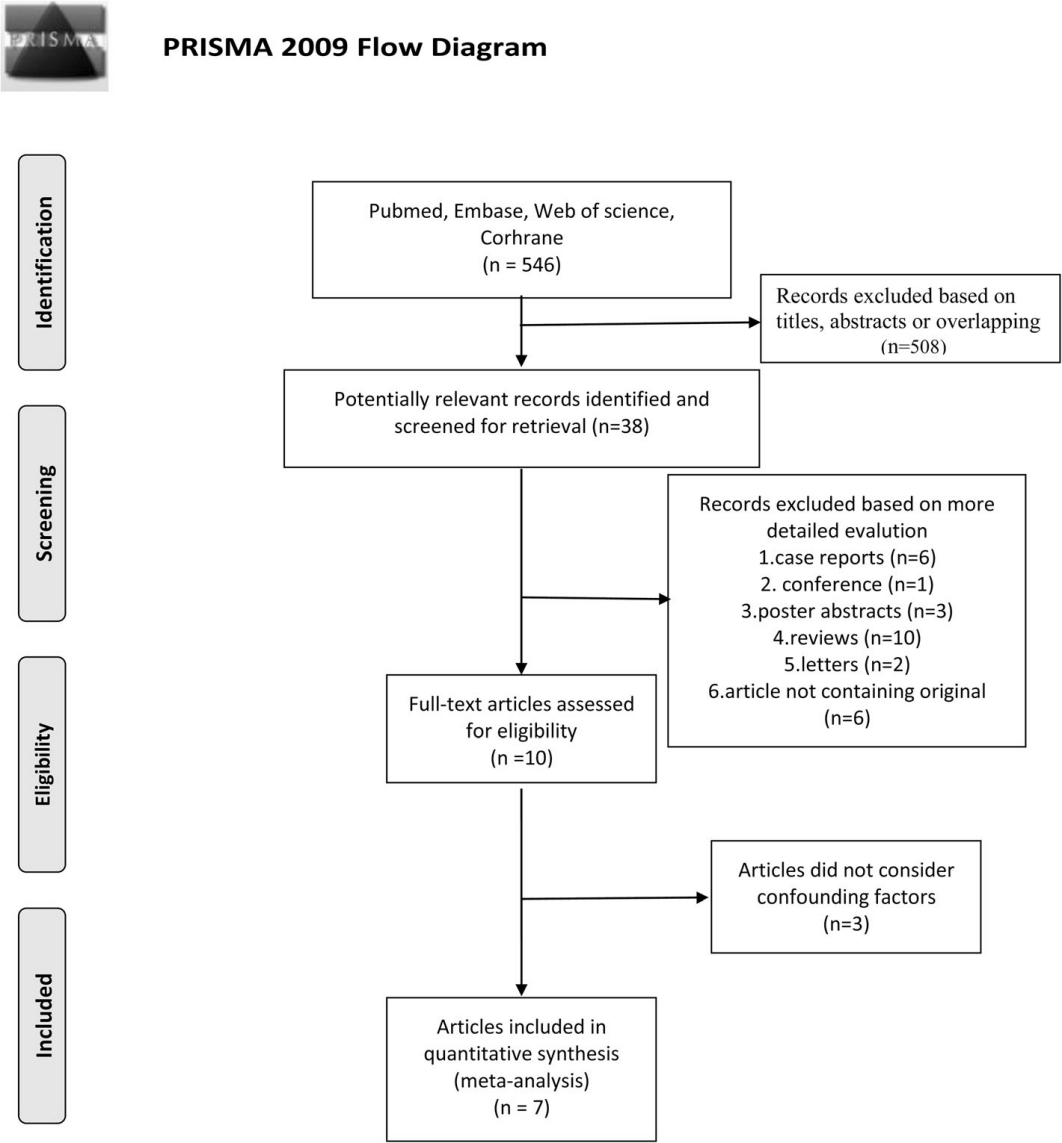
Summary of the studies selection process

A total of 546 relevant studies were retrieved by the initial literature search. The number of studies excluded by title and abstract screening was 508, and this was mainly because they were unrelated studies. The remaining studies were further assessed by 3 authors independently according to inclusion and exclusion criteria. Finally, 7 studies were eligible for inclusion in this meta-analysis (Fig. 1) [20–26]. No comparative randomized clinical trial was reported in the literature.

### Characteristics of the included study

The seven studies involved 5392 patients with only CCCPR and 3156 patients with OCCPR. The characteristics of all studies are shown in Table 1. These studies were published from 1993 to 2017. All seven reports were observational studies. No comparative randomized clinical trial was reported in the literature. Notably, most patients underwent OCCPR after CCCPR failure.

**Table 1:** Descriptions of included studies

Study (year)	Country/ Study period	Study design	Age (y)	Cause of arrest (n)		ROSC^a^		Survival to discharge	
				OCCPR^b^	CCCPR^c^	OCCPR^b^	CCCPR^c^	OCCPR^b^	CCCPR^c^
Masaya (1993) [20]	Not available (1986.1–1992.11)	A Prospective observational study	≥18	Non-trauma:26	Non-trauma:69	15/26	21/69	3/26	1/69
Azad (1994) [21]	American (1987.7–1991.7)	A retrospective study	<18	trauma:15	trauma:12	3/15	2/12		
Anastasia (1998) [22]	Greece (1993.12–1996.3)	A Prospective observational study	≥18	trauma:16	trauma:13	14/16	13/13		
Yoshihiro (2011) [26]	Japan (2001–2011)	A Case series observational study	<18 And ≥18	trauma:407	trauma:70	130/407	31/70		
Kodai (2016) [23]	Japan (2004.1–2012.12)	A retrospective cohort study	≥18	trauma:484	trauma:893			9/484	84/893
Matthew (2016) [24]	American (2014.4–2014.12)	A Prospective observational study	≥18	trauma:16	trauma:17	4/16	7/17		
Akira (2017) [25]	Japan (2004.1–2015.12)	A retrospective cohort study	≥18	trauma:2192	trauma:4318			40/2192	156/4318

^a^ Return of spontaneous circulation;

^b^ Open-chest cardiopulmonary resuscitation;

^c^ Closed-chest cardiopulmonary resuscitation

### Quality assessment

The quality features of the 7 studies are shown in Table 2. All 7 studies were of high quality.

**Table 2:** Quality assessment according to the Newcastle-Ottawa Scale

Study (year)	Masaya 1993 [20]	Azad 1994 [21]	Anastasia 1998 [22]	Yoshihiro 2011 [26]	Kodai 2016 [23]	Matthew 2016 [24]	Akira 2017 [25]
Selection	5	4	4	4	4	5	5
Comparability	1	1	1	1	1	1	1
Outcome	2	1	3	2	2	2	2
Total	8	6	8	7	7	8	8

### Primary outcome

**Fig. 2:**
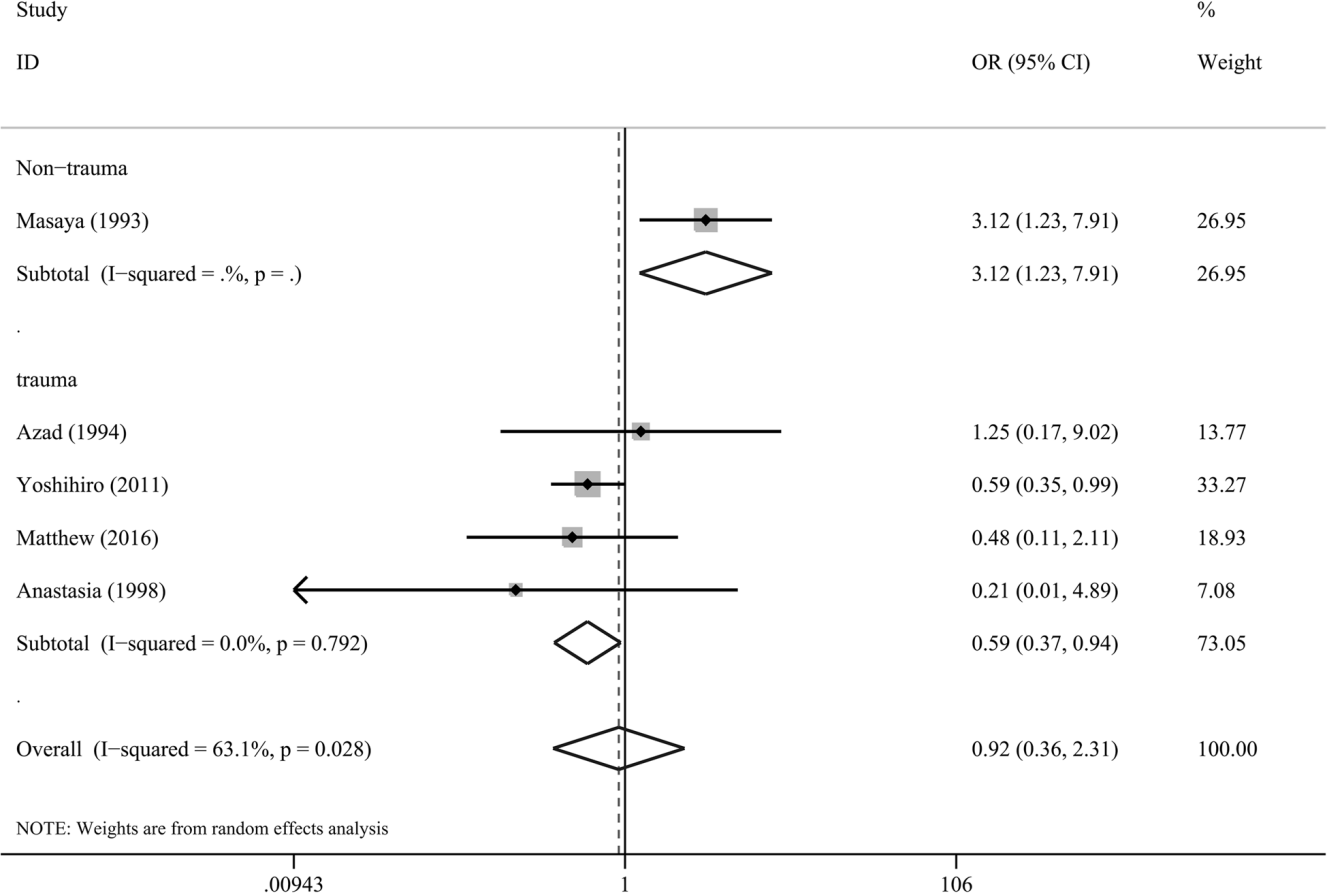
Forest plot of studies reporting ROSC

ROSC was reported in five studies (*n* = 661). The random effects model was used because of I^2^ = 63.1%. There was no significant difference in ROSC between OCCPR and CCCPR in cardiac arrest patients (OR = 0.92, 95%CI 0.36–2.31, *P* > 0.05, Fig. 2).

And subgroup analysis of cardiac arrest patients with trauma showed that CCCPR was associated with higher ROSC compared with OCCPR. The subgroup analysis of odds ratio (OR) were 0.59 (95%CI 0.37–0.94, *P* < 0.05) and the I^2^ were 0% (Fig. 2**)**.

### Secondary outcomes

**Fig. 3:**
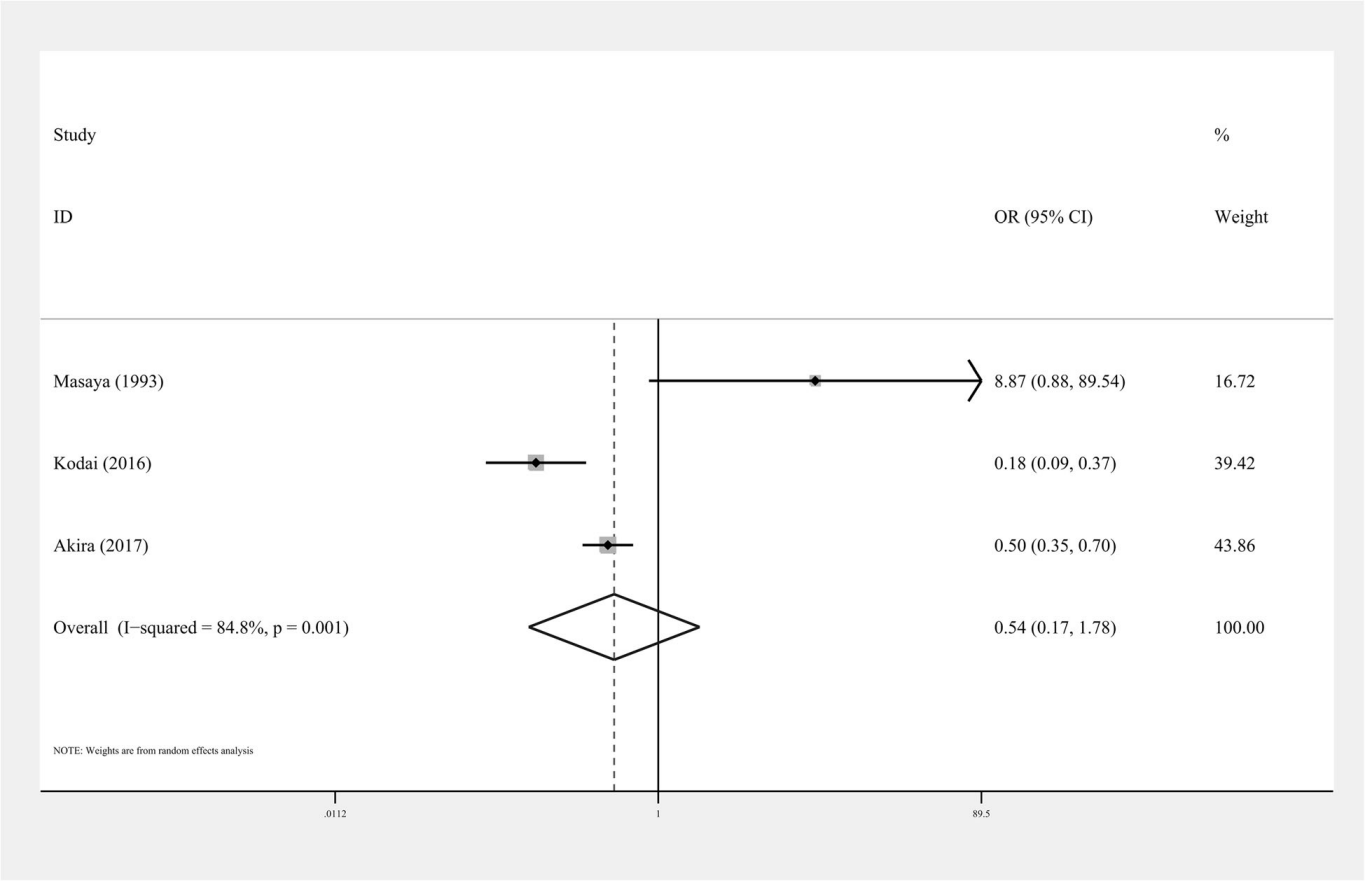
Forest plot of studies about survival to discharge

Three studies provided data regarding survival to discharge (*n* = 7982). The data were also analyzed by the random effects model according to heterogeneity test results (I^2^ = 84.8%). Overall, there was no significant difference in survival to discharge between OCCPR and CCCPR in cardiac arrest patients (OR = 0.54, 95%CI 0.17–1.78, *P* > 0.05, Fig. 3).

### Publication Bias

**Fig. 4:**
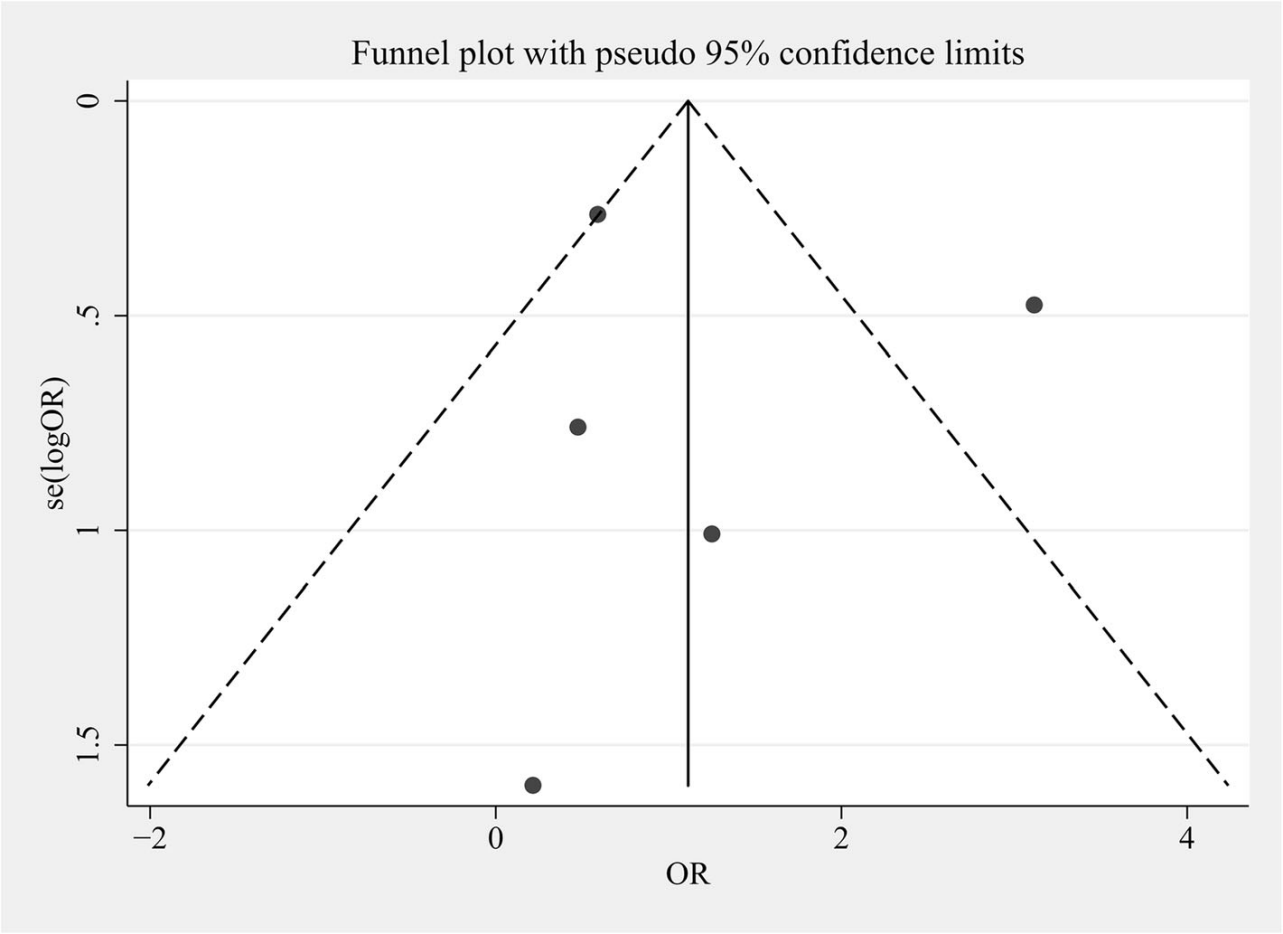
Funnel plot based on adds ratio for ROSC

The funnel plot showed that the publication bias of the meta-analysis (review) was acceptable (Fig. 4). No obvious publication bias was revealed by Begg’s asymmetry (*P* > 0.05).

## Discussion

Although the success rate of CCCPR is much lower than expected, and there is growing evidence that OCCPR is superior to CCCPR [13], no meta-analysis assessed superiority between OCCPR and CCCPR. This is the first meta-analysis which evaluates the effect of OCCPR and CCCPR.

As we all know, the primary goal of CPR is ROSC, and the ultimate goal is to improve survival until discharge. Therefore, we retrieved 7 studies to comparatively assess ROSC and survival to discharge in OCCPR and CCCPR. Overall, there were no significant differences in ROSC and survival to discharge between the OCCPR and CCCPR groups. Subgroup analysis of cardiac arrest patients with trauma showed that CCCPR was associated with higher ROSC compared with OCCPR. And subgroup analysis of cardiac arrest patients with non-trauma showed that OCCPR was associated with higher ROSC compared with CCCPR.

Astonishingly, our overall findings differ from a previous systematic review performed in 2008 claiming that OCCPR is superior to CCCPR [13]. In addition, the latter study also showed that the superiority of OCCPR was consistently demonstrated by over 18 good quality animal studies [13]. The discrepancy may be explained by the following reasons. Firstly, it is worth noting that in the current meta-analysis, OCCPR was mostly performed after failure of CCCPR. Meanwhile, time to perform CCCPR before OCCPR was from 2 min to more than 2 h. Current guidelines emphasize early identification, and early chest compression with minimal interruptions, and timely advanced cardiac life support [27]. The above also applies to OCCPR. In addition, human studies showed that a short CCCPR time before OCCPR is significantly associated with higher ROSC rates [28]. Studies have showed that success rates can increase to 80% in cardiac arrest patients who performed OCCPR immediately [22, 29]. In the present meta-analysis, OCCPR in the vast majority of patients was performed after failure of CCCPR, and CCCPR duration varied from 2 min to 2 h, which may greatly reduce the success rate of OCCPR. Secondly, patients in the previous systematic review of 2008 suffered from cardiac arrest following cardiac surgery [13]. A human study conducted by Rhee et al. showed that the survival rate of trauma patients is highest with the injury site located in the heart [30].

Subgroup analysis of cardiac arrest patients with trauma showed that CCCPR was associated with higher ROSC compared with OCCPR. Most of the trauma patients included in this study were blunt trauma patients. Another systematic review in 2013 indicated that survival rate in patients with blunt trauma treated by emergency department thoracotomy is very low; therefore, some authors suggested that OCCPR should be avoided in case of blunt trauma without vital signs on admission [31]. Some studies also showed that the survival rate of patients with blunt trauma is only 1.4% [30]. The Western Trauma Association also points out that the survival rate for non-vital blunt trauma patients is less than 1% [17]. Perhaps because the majority of trauma patients included in this meta-analysis were blunt trauma patients, which dominated the results. There are also not enough patients with penetrating trauma included, and more study is needed for further evaluation. However, for trauma patients who cannot perform CCCPR, such as chest trauma, rib fracture, flail chest and other chest injuries, OCCPR should be considered.

Subgroup analysis of cardiac arrest patients with non-trauma showed that OCCPR was associated with higher ROSC compared with CCCPR. Direct cardiac massage can produce higher coronary perfusion pressure and elevated systemic flow compared with indirect cardiac massage. Moreover, many reliable human studies have shown that OCCPR has a higher success rate than CCCPR. For example, in 1953, Stephenson et al. published cardiac arrest data from 1200 OCCPR patients with a recovery rate of 28% [32]. Subsequently, Briggs et al. reported patients with cardiac arrest during a 30-year period. In this study 58% of patients who underwent OCCPR within 4 min recovered without impaired neurological function [33]. In 2011, Karhunen et al. reported 76 patients who had cardiac arrest, with 62 (82%) survivors recorded after immediate OCCPR [29].

The above findings indicated that OCCPR may achieve effect in patients with cardiac arrest. But OCCPR is invasive, and OCCPR cannot be performed anytime and anywhere. It is also difficult to extensively train emergency doctors to perform emergency thoracotomy and internal defibrillation. And, there is also the issue of having the proper equipment readily available. In contrast, CCCPR can be performed anywhere, anytime. CCCPR is more convenient, easier and less costly than OCCPR. however, for cardiac arrest patients who cannot perform CCCPR, such as patients with rib fracture, flail chest and open-heart surgery, they can decisively choose to implement OCCPR.

This was a complex meta-analysis, because OCCPR in the vast majority of patients was performed after failure of CCCPR. As a result, this meta-analysis was actually evaluated superiority between OCCPR and only CCCPR. Meanwhile, in order to distinguish the effects between OCCPR and CCCPR for patients with traumatic and non-traumatic cardiac arrest respectively, we divided patients with traumatic cardiac arrest and non-traumatic cardiac arrest into subgroups and compared the effects between OCCPR and CCCPR in terms of ROSC.

In conclusion, for patients with cardiac arrest, we should implement CCCPR as soon as possible. However, for cardiac arrest patients with chest trauma who cannot perform CCCPR, OCCPR should be implemented as soon as possible. In a word, we should individualize the choice of cardiopulmonary resuscitation methods.

### Limitations of the review

In the present study, there were some limitations. First, the included studies were observational trials, so selection bias could be introduced. Secondly, no age restriction was adopted for participants. Animal experiments suggested that pediatric CCCPR is more effective than adult CCCPR due to differences in rib shape and elasticity of the chest wall cavity [34]. We attempted to conduct subgroup analysis to address this issue, but the number of studies evaluating each endpoint was limited. Thirdly, the implementation of CCCPR prior to OCCPR may weaken the effectiveness of OCCPR. Fourthly, there were very limited published data about comparing OCCPR and CCCPR. These limitations might also contribute to the extreme heterogeneity of the retrieved studies.

## Data Availability

All data generated or analyzed during this study are included in this published article.

## Abbreviations

CCCPR: Closed-chest cardiopulmonary resuscitation
CI: Confidence interval
MOOSE: Meta-analysis Of Observational Studies in Epidemiology
NOS: Newcastle-Ottawa Scale
OCCPR: Open-chest cardiopulmonary resuscitation
OR: Odds ratio
PRISMA: Preferred Reporting Items for Systematic Reviews and Meta-Analysis
ROSC: Return of spontaneous circulation
